# Identification and Expression of *Capa* Gene in the Fire Ant, *Solenopsis invicta*

**DOI:** 10.1371/journal.pone.0094274

**Published:** 2014-04-09

**Authors:** Man-Yeon Choi, Rene Köhler, Robert K. Vander Meer, Susanne Neupert, Reinhard Predel

**Affiliations:** 1 United States Department of Agriculture-Agricultural Research Service (USDA-ARS), Center of Medical, Agricultural and Veterinary Entomology (CMAVE), Gainesville, Florida, United States of America; 2 Zoological Institute, Biocenter University of Cologne, Cologne, Germany; Goethe University Frankfurt, Germany

## Abstract

Recent genome analyses suggested the absence of a number of neuropeptide genes in ants. One of the apparently missing genes was the *capa* gene. *Capa* gene expression in insects is typically associated with the neuroendocrine system of abdominal ganglia; mature CAPA peptides are known to regulate diuresis and visceral muscle contraction. The apparent absence of the *capa* gene raised questions about possible compensation of these functions. In this study, we re-examined this controversial issue and searched for a potentially unrecognized *capa* gene in the fire ant, *Solenopsis invicta*. We employed a combination of data mining and a traditional PCR-based strategy using degenerate primers designed from conserved amino acid sequences of insect *capa* genes. Our findings demonstrate that ants possess and express a *capa* gene. As shown by MALDI-TOF mass spectrometry, processed products of the *S. invicta capa* gene include three CAPA periviscerokinins and low amounts of a pyrokinin which does not have the C-terminal WFGPRLa motif typical of CAPA pyrokinins in other insects. The *capa* gene was found with two alternative transcripts in the CNS. Within the ventral nerve cord, two *capa* neurons were immunostained in abdominal neuromeres 2–5, respectively, and projected into ventrally located abdominal perisympathetic organs (PSOs), which are the major hormone release sites of abdominal ganglia. The ventral location of these PSOs is a characteristic feature and was also found in another ant, *Atta sexdens*.

## Introduction

Although higher insect taxa (“nsect order” diverged more than 200 mya, general features of the hexapod neuroendocrine system, including cell location, hormone release sites, and sequences of the peptide hormones are strongly conserved. This conservation reflects the functional significance of these peptide messenger molecules. Therefore, it was unexpected that recent genome analyses failed to identify several neuropeptide genes in a number of insects. For the key substances of the segmentally arranged perisympathetic organs (PSOs), which store products of the *extended fmrf* gene in the thorax and products of the *capa* gene in the abdomen, this situation was first described from the parasitic wasp *Nasonia vitripennis* (Hymenoptera; [1]). Here, the absence of otherwise highly conserved peptide hormones could be attributed to the miniaturization of the nervous system or parasitic lifestyle but later it was described that ant genomes do not possess *fmrf* and *capa* genes as well [2].

We addressed this question with a simple approach and used a CAPA antiserum raised against a cockroach CAPA peptide to screen the ventral nerve cord (VNC) of the fire ant, *Solenopsis invicta*, for CAPA immunoreactive (ir) cells. Indeed, we observed the typical CAPA immunoreactivity in neurons of abdominal ganglia and identical cells were immunostained in other ant species as well. These findings were the reason to search for the potentially overlooked *capa* gene from *S. invicta*. To achieve this aim, we employed a PCR-based strategy using degenerate primers designed from conserved amino acid sequences of *capa* genes, and searched for similar genes from the fire ant genome [3]. In the present study we demonstrate the identification, structure and characterization of the *capa* gene, transcription profiles, translated mature peptides, and location of *capa* neurons in the abdominal VNC and attached release sites. The distribution of *capa* neurons in the VNC of *S. invicta* is compared with that in another ant, *Atta sexdens*, and the honey bee, *Apis mellifera*.

## Material and Methods

### 1 Insects

Fire ants, *S. invicta*, were taken from monogyne (single egg laying queen) colonies collected in the Gainesville area in Florida, USA. Colonies collected by excavation were removed from the soil by floating out the workers and brood as previously described [4]. No specific permits were required for the described field collections and the collections did not affect endangered or protected species. All colonies were fed crickets and 10% sugar solution absorbed onto wads of tissue and maintained under standard laboratory conditions. Leaf-cutter ant, *A. sexdens*, was provided by Wolfgang Rössler (Würzburg, Germany), and *A. mellifera* was provided by Elke Woker (Jena, Germany). All animal procedures were conducted in compliance with protocols approved by local government authorities and were in accordance with National Institutes of Health guidelines.

### 2 Molecular cloning and characterization

Poly (A)^+^ RNA was isolated from winged females without head by Micro Fast mRNA purification kit (Invitrogen, CA, USA) and used to synthesize cDNA with the GeneRacer cDNA synthesis kit (Invitrogen). First we designed degenerate primers based on conserved amino acid sequences (MWFGPRLG) for sense primers, 5′-ATGTGGTTYGGNCCNMGNYTNGG-3′ and 5′-TGGTTYGGNCCNMGNYTNGG-3′, and antisense primers, 5′-CCNARNCKNGGNCCRAACCACAT-3′ and 5′-CCNARNCKNGGNCCRAACCA-3′. Those primers were used for PCR amplification with cDNA synthesized from winged females without head using 5′ and 3′-Rapid Amplification of cDNA End (RACE) method (Invitrogen) using manufacturer's procedure. Secondly, BLAST was employed to search the *S. invicta* genome [3] with known insect *capa* genes from GenBank. Specific forward and reverse primers, 5′-TAC ACTCCAAGA CTAGGTCGCGAGAGTTGA-3′ and 5′-TCA ACTCTCGCG ACCTAGTCTTGGAGTGTA-3, were designed based on the amino acid sequence YTPRLGRESE found in the *S. invicta* genome. These primers were used for 3′- and 5′- RACE, respectively, and PCR amplification using the manufacturer's procedure (Invitrogen). RACE PCR products were checked using 1.5% agarose gel electrophoresis and visualized using GelRed (Biotium, CA, USA) under a UV light. The PCR products were then purified, cloned into a subcloning vector (TOPO-TA, Invitrogen), sequenced by Sanger DNA sequencing (Interdisciplinary Center for Biotechnology Research, ICBR, University of Florida, Gainesville, FL, USA), and analyzed by Genetyx DNA software (ver. 10, Genetyx Co., Tokyo, Japan). Resulting sequences were used to design another primer set: 5′-CACTGGTCACAGAAATGCAGGACAACCGG-3′ and 5′-TGGGTCGCCTATGCAAATAGGAGAAAACG-3′ to amplify a full sequence of the fire ant *capa* gene. PCR was performed for 35 cycles at 95°C for 30 s, 63°C for 30 s, and 72°C for 1 min, then 72°C for 5 min using iTaq DNA polymerase (Bio-Rad, CA, USA). PCR products after gel purification were inserted into the subcloning vector described above and analyzed by DNA sequencing. We found a∼7-Kb long DNA sequencing contig containing exons and introns of the *capa* gene (see Figure S1) in the *S. invicta* genome data using the obtained *capa* mRNA sequence and analyzed by Genetyx DNA software.

### 3 Reverse transcriptase (RT)-PCR for *capa* expression

Total RNA was isolated from the following fire ant samples: fertilized eggs, female larvae, female pupae, and workers without head; in addition head, brain, subesophageal ganglion (SEG), and VNC of workers, using the PureLinkTM RNA Kit (Invitrogen). RNA was quantified by a NanoDrop 2000 (Thermo Scientific, PA, USA) and used to synthesize cDNA using SuperScript RTIII according to the manufacturer's protocol (Invitrogen). The first-strand cDNA synthesized from the different tissues was used for PCR amplification with a primer set: 5′-CACTGGTCACAGAAATGCAGGACAACCGG-3′and 5′-TGTACCCTCCTTGACCTTGACTA TTTCG-3′ for *capa*, and a primer set, 5′-AGCGACGTGTCCGAGATGATCGTCACCAG-3′ and 5′-CT AACTCGGATAGTTGCCTATAGTTTTCG-3′ for *pyrokinin/pheromone biosynthesis activating neuropeptide* (*pk/pban*). A fragment of 100 nucleotides of the fire ant 18S rRNA was amplified as positive control as described previously [5]. PCR was performed as follows: 35 cycles at 95°C for 30 s, 55°C for 30 s, and 72°C for 50 s, then 72°C for 5 min using iTaq DNA polymerase (Bio-Rad). Prepared PCR products were checked using 1.5% agarose gel electrophoresis, purified and the sequences confirmed by DNA sequencing as described above.

### 4 Mass spectrometry

#### Dissection and sample preparation for mass spectrometry

Immobilized large workers and/or reproductives of *S. invicta* were fixed with insect pins and completely submerged in insect saline (in mM: NaCl 126, KCl 5.4, NaH_2_PO_4_ 0.17 and KH_2_PO_4_ 0.22, pH 7.4). Subsequently, the thorax and abdomen (including the petioles) were dorsally opened with an ultrafine scissor and, after removal of the gut, the VNC was dissected and transferred into a separate dish containing saline. The tiny abdominal PSOs were visualized and manually dissected with a high resolution stereomicroscope (Lumar V12; Carl Zeiss, Göttingen, Germany), and transferred with a glass capillary in a drop of purified water on the sample plate for MALDI-TOF mass spectrometry. The water was removed using the transfer glass capillary before the dried samples were covered with a mixture of 0.2 μl α-cyano-4-hydroxycinnamic acid (CHCA, Sigma-Aldrich, Steinheim, Germany) dissolved in 50% methanol. After drying at room temperature, each spot was finally rinsed with water for a few seconds.

#### Matrix-assisted laser desorption ionization time-of-flight (MALDI-TOF) mass spectrometry

Mass spectrometric analysis was performed on an ABI 4800 proteomics analyzer (AB Sciex, Darmstadt, Germany). The acquisitions were taken in manual mode. Instrument calibration was performed using the peptide standard kit 1 (Bruker Daltonik GmbH, Bremen, Germany). Initially, the instruments were operated in reflectron mode, in order to determine the parent masses. For the tandem MS experiments, we used PSD as well as CID mode. The fragmentation data obtained in these experiments were analyzed with Data Explorer 4.3 (AB Sciex) and used to confirm the sequences of the fire ant neuropeptides [6].

### 5 Immunocytochemistry and documentation

VNCs of adults were fixed for 3 h at 4°C with 4% paraformaldehyde in phosphate-buffered saline (PBS), pH 7.2. Subsequently, samples were washed 3 times for 15 min in PBS 1% Triton X-100 at room temperature. The preparations were then incubated for 2 days at room temperature in anti-CAPA-periviscerokinin-2 (PVK-2) serum (1∶4000), which was raised against *Periplaneta americana* CAPA-PVK-2 (GSSSGLISMPRV-NH_2_) [7], diluted with PBS 1% Triton X-100 containing 0.25% bovine serum albumin and 10% normal goat serum. Following washing in PBS 1% Triton X-100 for 24 h, the preparations were incubated with the secondary antibody conjugated to Cy3 (1∶3000; Jackson ImmunoResearch, Suffolk, UK) in PBS for 2 days at room temperature. Finally, the preparations were washed again for 24 h in PBS 1% Triton X-100 and mounted in Mowiol (Calbiochem, Darmstadt, Germany). Immunostainings were examined with a confocal laser scanning microscope (ZEISS LSM 510 Meta system, Jena, Germany), equipped with a Plan-Neofluar 40x/1.3 oil objective and a HeliumNeon1 laser (wavelength 543 nm). Serial optical sections were assembled into combined images. Images were exported and processed (contrast, brightness, image detail, file format) with Adobe Photoshop 7.0 software. For the scale drawings, printouts of the LSM scans were traced onto tracing paper using a technical pen. For a correct assignment of the 3D position of immunostained cells within the ganglion, preparations were examined with a fluorescence stereomicroscope (Lumar V12) during drawing.

## Results

### 1 *Capa* mRNA and precursor sequences

**Figure 1:**
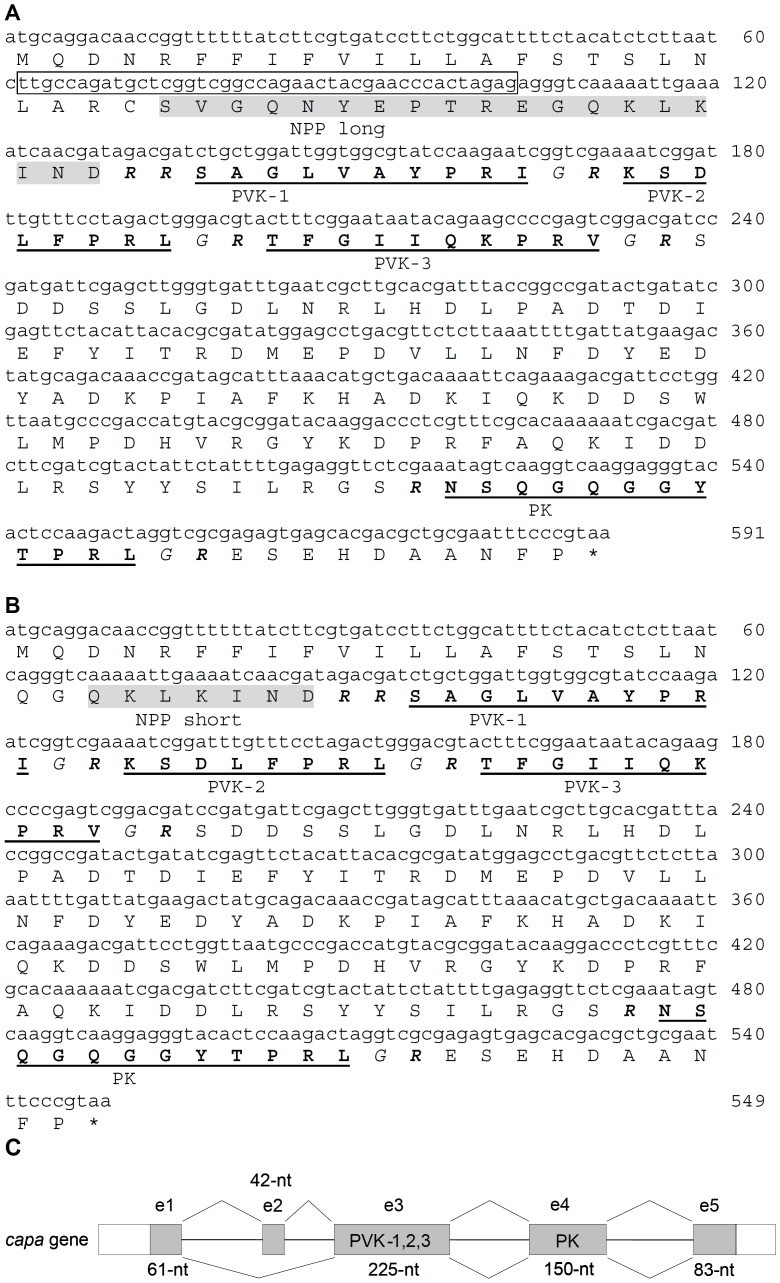
*S. invicta capa* mRNA transcripts, translated amino acids, and schematic overview of the two splice variants. **A**) Long transcript. **B**) Short transcript. *S. invicta* PVK-1, −2, −3, and PK neuropeptides (underlined) are predicted using putative endoproteolytic cleavage sites (bold italic); Gly as potential amidation signal is italicized. The sequence between the signal peptide and PVK-1 is indicated as N-terminal precursor peptide (NPP; grey shaded). **C**) The 5-exon (e) *capa* gene is alternatively spliced in two transcripts; exon 2 is skipped in the short transcript. The sequence of the *capa* gene including exons and introns is given in the File S1. GenBank accession numbers of the mRNA transcripts are KJ020267 (long form) and KJ020268 (short form).

Two splice forms of *S. invicta capa* mRNA were identified (Fig. 1). The long transcript (591 nucleotides) and the short transcript (549 nucleotides) encode 196 amino acids and 182 amino acids, respectively. Both proteins contain six potential endoproteolytic cleavage sites [8], [9], R^44^-R^45^, R^57^, R^67^, R^79^, R^172^, R^186^ (Fig. 1A; numbering refers to the long transcript). Four of the predicted mature peptides have a C-terminal amide group provided by glycine. Three of these peptides, SAGLVAYPRI-NH_2_, KSDLFPRL-NH_2_, and TFGIIQKPRV-NH_2_ are considered putative CAPA-PVKs, and NSQGQGGYTPRL-NH_2_ shows sequence similarity with PK peptides. The C-terminal motif WFGPRL-NH_2_, which is typical of CAPA-PKs in most insects studied so far [10], is not present in the CAPA precursor sequence.

### 2 *Capa* gene structure

*S. invicta capa* pre-mRNA forms alternative RNA splice variants to generate two transcripts that are a long form incorporating five exons and a short form incorporating four exons from ∼40 kilo-nucleotide (nt) entire genomic DNA (see Figure S1). The splice variants show a 42-nt difference but the predicted mature CAPA-PVK/PK peptides are not different (Fig. 1). The three PVKs and the PK of the fire ant *capa* gene are encoded on exon 3 and 4. Exon 2 (42-nt) is skipped by alternative 5′ splicing in the short form where an intron gap (∼2-Knt) exists between exons 1 and 3 (Figs. 1C and S1).

### 3 *Capa* gene transcription

**Figure 2:**
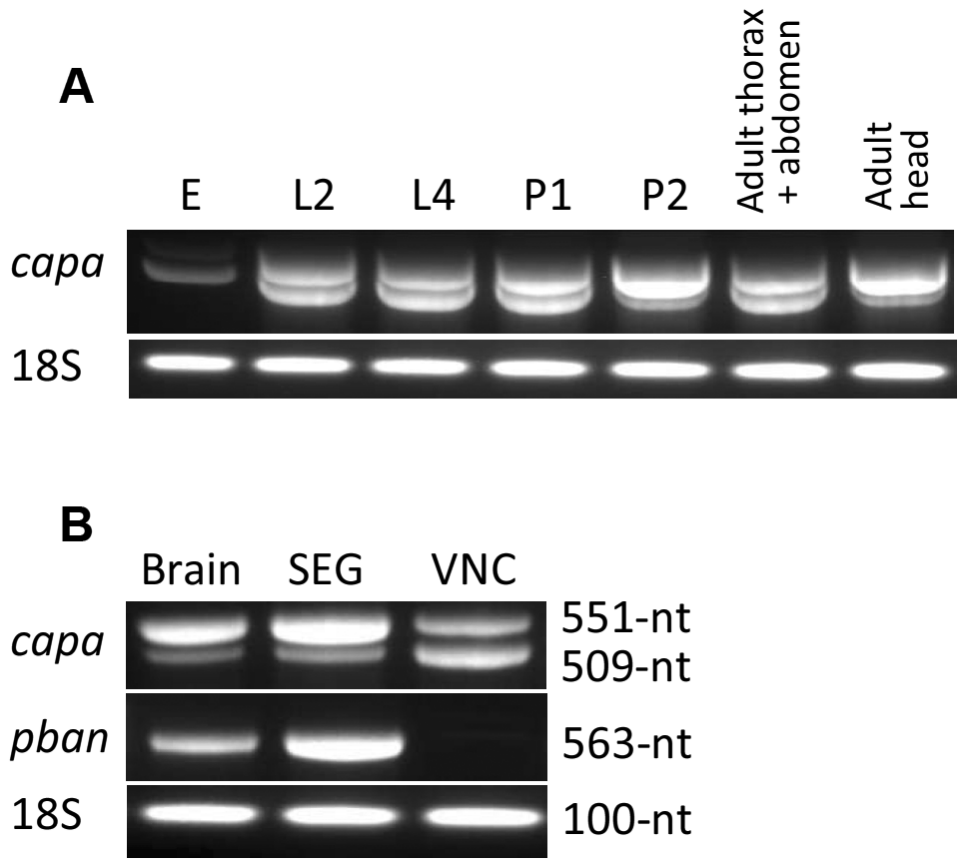
Expression profiles of *S. invicta capa* mRNA in different developmental stages (A) and different parts of CNS from winged females (B). The analysis of *capa* mRNA expression was complemented by the analysis of *pk/pban* mRNA expression in CNS samples of winged females (B). 1st cDNA and 35 cycles of PCR; *S. invicta* 18S gene was used as positive control. E, embryonic egg; L2/L4, second/fourth instar larva; P1/P2, early/late pupa; SEG, subesophageal ganglion from winged females; VNC, ventral nerve cord from winged females.

*Capa* mRNA was expressed in all developmental stages which have been studied (Fig. 2) but the transcription level was weak in the embryo. A particularly strong expression of *capa* mRNA was found in late pupae and adult insects. Both mRNA transcripts were detected in the CNS from larvae to adults while only the long form was observed in embryonic (egg) tissue. The ratio of both transcripts changed during the postembryonic development. In larval and early pupal stages as well as in headless adult ants, the expression of the two RNA forms was rather similar. In late pupae and adult head samples, the long form was found to be more abundant (Fig. 2A). A comparison of *capa* gene transcription in different parts of the CNS of adult females indicated that the long transcript is more abundant in brain and SEG whereas the short form is slightly more abundant in abdominal ganglia (Fig. 2B). A single form of *pk/pban* mRNA was observed in brain and SEG but not in the VNC (Fig. 2B).

### 4 Identification of *capa* gene products by mass spectrometry

**Figure 3:**
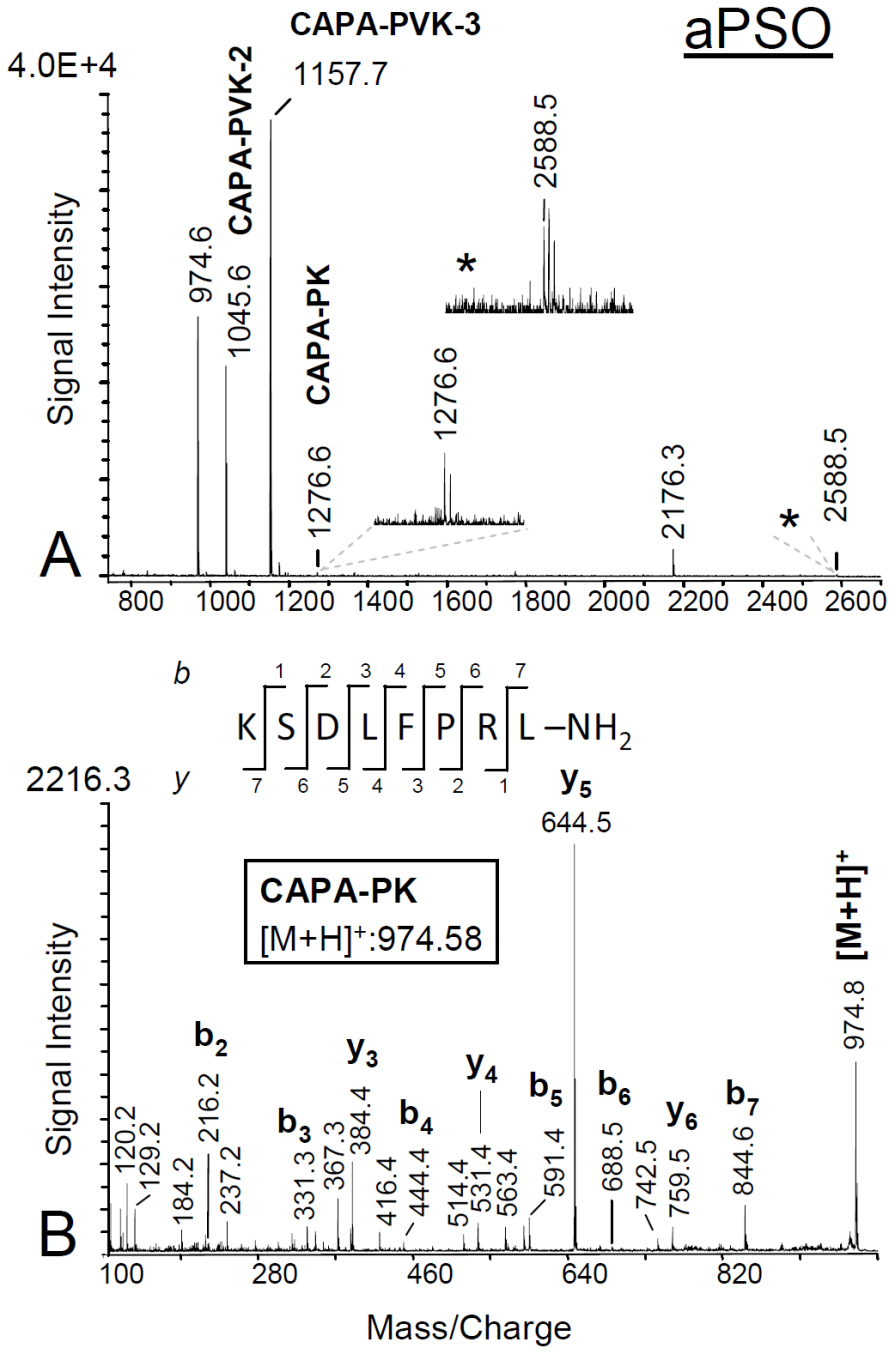
MALDI-TOF mass spectra from a preparation of a single abdominal PSO of female *S. invicta* (direct tissue profiling). A) Mass fingerprint spectrum (*m/z* 750–2700); prominent signals indicating the presence of three PVKs and the NPP (long transcript form) are detectable. The predicted PK shows a very weak signal intensity. This low signal intensity is likely a result of incomplete cleavage from the remaining C-terminus of the precursor sequence. With the exception of the supposed CPP at *m/z* 2588.5, sequences of all designated peptides were confirmed by MS/MS analyses. B) MALDI MS/MS fragment spectrum of the ion signal at *m/z* 974.6 (see 3A) under conditions of CID off. Fragment series (b- and y-type ions are labelled) confirmed the sequence of *S. invicta* PVK-2. aPSO, abdominal perisympathetic organ; PVK, periviscerokinin; PK, pyrokinin; CPP, C-terminal precursor peptide; NPP, N-terminal precursor peptide.

Direct tissue profiling of single abdominal PSOs of adult *S. invicta* yielded highly reproducible mass spectra in the mass range of *m/z* 750–2700 (Fig. 3A). The most prominent ion signals indicated the presence of the three predicted CAPA-PVKs and N-terminal precursor peptide (NPP) of the long transcript, preceding the CAPA-PVK-1 sequence (SVGQNYEPTREGQKLKIND-OH; see Table 1). The predicted NPP of the short transcript sequence (pQ/QKLKIND-OH) was not observed. Sequences of PVKs and NPP of the long transcript were confirmed by fragment analyses (see Fig. 3B for CAPA-PVK-2). In contrast to the PVKs and NPP, the predicted PK (NSQGQGGYTPRLa) was identified in mass spectra with very low ion signal intensity. However, a substance mass similar with a hypothetical C-terminal precursor peptide (CPP), consisting of PK and the remaining C-terminal amino acids from the CAPA precursor (NSQGQGGYTPRLGRESEHDAANFP-OH), was detected. Ion signal intensity of this peptide was not sufficient for MS/MS experiments.

**Table 1:** List of peptides from *capa* and *pk/pban* genes of *S. invicta*, identified by MALDI-TOF/TOF mass spectrometry in this study.

peptide name	sequence	m/z	aPSO	CC
**CAPA-peptides**				
long NPP	SVGQNYEPTREGQKLKIND-OH	2176.10	x	-
PVK-1	SAGLVAYPRI-NH_2_	1045.62	x	-
PVK-2	KSDLFPRL-NH_2_	974.58	x	-
PVK-3	TFGIIQKPRV-NH_2_	1157.72	x	-
PK	NSQGQGGYTPRL-NH_2_	1276.64	x	-
CPP	NSQGQGGYTPRLGRESEHDAANFP-OH	2588.19	x^1^	x^1^
**PK/PBAN**				
PK-1	TSQDIASGMWFGPRL-NH_2_	1664.82	-	x
PK-2	pQPQFTPRL-NH_2_	968.53	-	x
PK-3	GSGEDLSYGDAYEVDEDDHPLFVPRL-NH_2_	2894.31	-	x
PK-4	RLPWIPSPRL-NH_2_	1233.76	-	x
CPP	pQLRNVLRKL-OH	1122.71	-	x^1^

CPP, C-terminal precursor peptide; NPP, N-terminal precursor peptide; PBAN, pheromone biosynthesis activating neuropeptide; PVK, periviscerokinin; PK, pyrokinin; aPSO, abdominal PSO; CC, *corpora cardiaca*.

1 mass match only.

**Figure 4:**
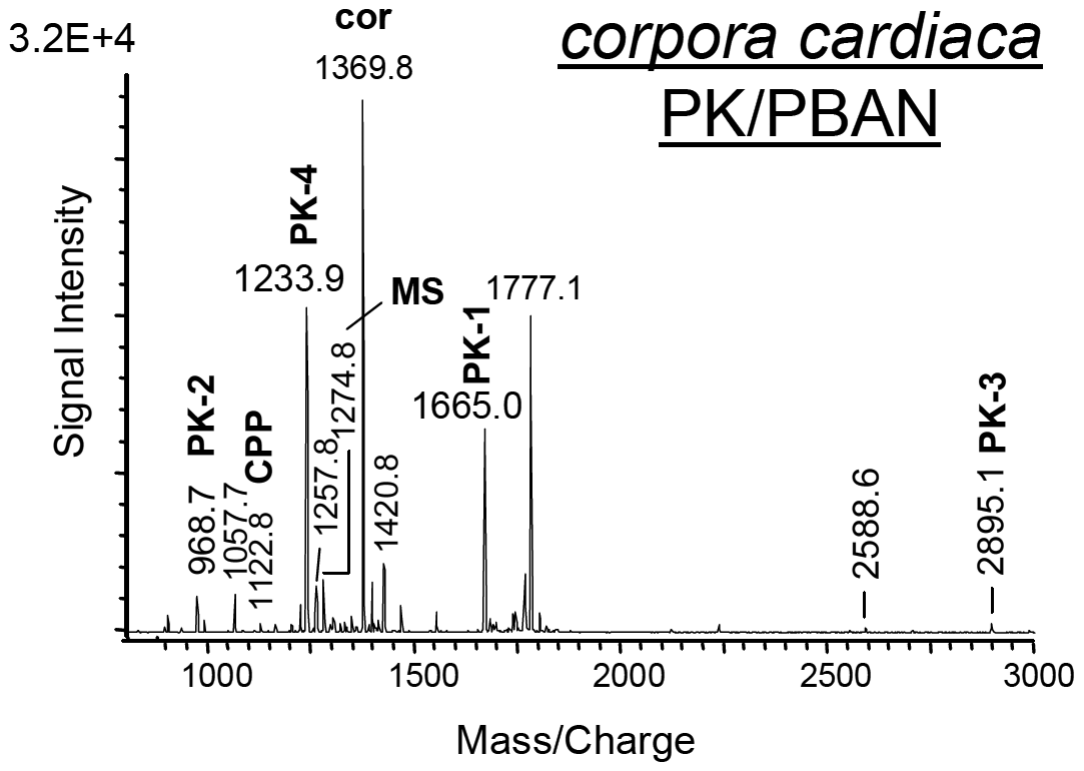
MALDI-TOF mass fingerprint spectrum (*m/z* 800–3000) from a preparation of *corpora cardiaca* from a female *S. invicta* (direct tissue profiling). Ion signals indicating the presence of products from the *pk/pban* gene are labeled; their sequences were subsequently confirmed by MS/MS experiments (not shown). The ion at *m/z* 2588.6 matched the predicted mass of CPP from the CAPA precursor but ion intensity of this peptide was not sufficient for MS/MS experiments. CPP, C-terminal precursor peptide (in the mass spectrum, CPP processed from the PK/PBAN precursor is designated); cor, corazonin; MS, myosuppressin (pQ/Q forms); PK, pyrokinin.

Due to C-terminal sequence similarity of products from the *capa* gene and products from the *pk/pban* gene, PVK and PK/PBAN antisera both recognize products of the two genes. In our experiments, no product of the *pk/pban* gene could be detected in mass spectra of abdominal PSOs and abdominal VNC. *Pk/pban* gene transcription was also not observed in the abdominal ganglia (see Fig. 2). Thus, PK/PBAN immunoreactivity in neurons of abdominal ganglia [5] can be attributed to the CAPA peptides. Conversely, mass spectra obtained from *corpora cardiaca* of *S. invicta* did not indicate the presence of CAPA-PVKs or PK from the *capa* gene. Instead, all predicted PKs [5] and the pyroglutamate form of the CPP from the *pk/pban* gene (pQLRNVLRKL-OH) were observed in mass spectra from *corpora cardiaca* (Fig. 4; Table 1).

### 5 Localization of CAPA immunoreactivity in the VNC of *S. invicta* and related Hymenoptera

#### 5.1 S. invicta

**Figure 5:**
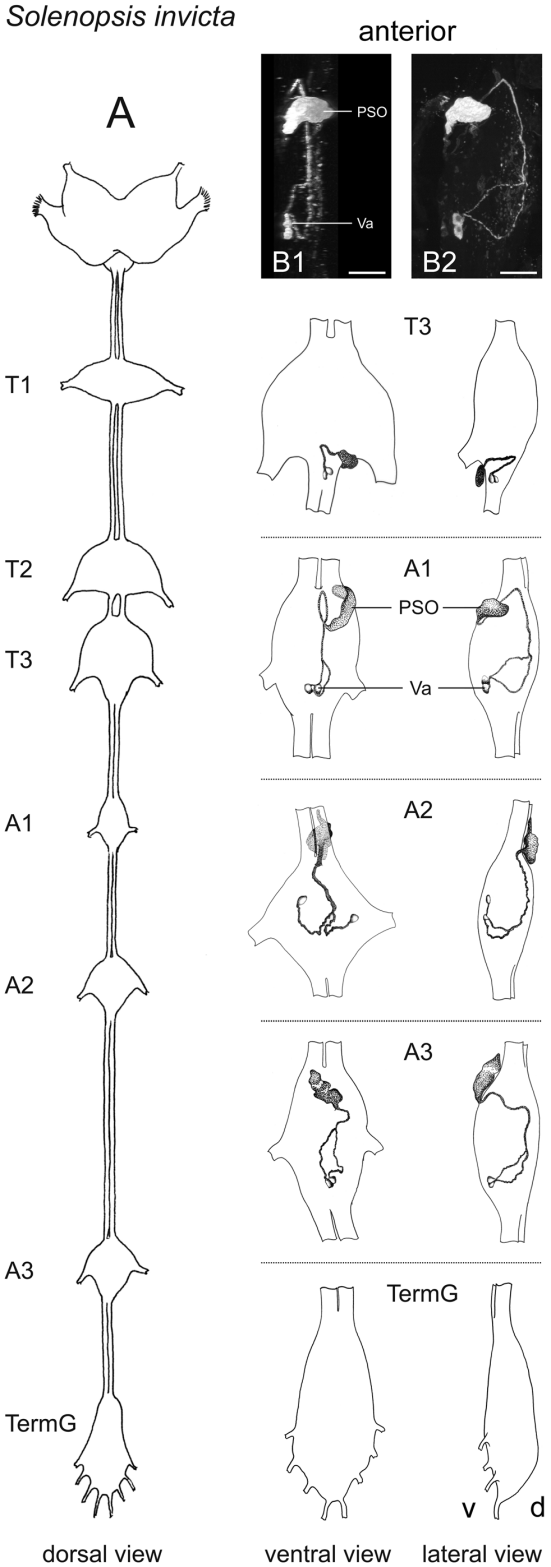
Localization of CAPA-ir neurons in the VNC of *S. invicta*. **A**) Schematic overview of the entire CNS (left) and ganglia of the VNC (right), showing CAPA-ir neurons with attached PSOs in abdominal neuromeres 2–5. Abdominal neuromeres 1 and 2 are fused with thoracic neuromere 3 in the metathoracic ganglion (T3). Therefore, the first unfused abdominal ganglion (A1) represents abdominal neuromere 3 and so on. Axons of the ventral *capa* neuron pairs (Va-cells) first project dorsally into the dorsal midline of the ganglia and then anteriorly. Subsequently, these fibers pass to the ventral side and enter ventrally located PSOs. In a few cases, PSOs were found in an anterodorsal position (see abdominal ganglion 2). In the terminal ganglion, no CAPA-ir cells were detected. **B**) CAPA immunostaining in the third unfused abdominal ganglion (A3); ventral (**B1**) and lateral view (**B2**). A, abdominal ganglion; d, dorsal; PSO, perisympathetic organ; T, thoracic ganglion; TermG, terminal ganglion; v, ventral; scale bar 25 μm.

The VNC of adult *S. invicta* consists of three thoracic ganglia with the ganglion in the metathorax composed of thoracic neuromere 3 and abdominal neuromeres 1+2, three unfused abdominal ganglia, and the terminal ganglion. The first two unfused abdominal ganglia are located in the first and second node of the petiole, respectively. Immunostainings with the *P. americana* PVK-2 antiserum yielded CAPA-ir neurons in abdominal neuromeres 2–5; somata of these cells were always located ventrally. Abdominal neuromere 1, which is generally fused with the metathoracic ganglion in pterygote insects [11], did not contain CAPA-ir cells. Abdominal neuromere 2 showed a bilateral pair of CAPA-ir neurons which cell bodies were located posteromedially. The axons of the CAPA-ir cells first projected together dorsomedially into the anterodorsal midline of the neuromere before passing to the ventral side and entering an anteroventrally located bulblike PSO. A similar projection of CAPA-ir neurons was found in the three unfused abdominal ganglia although the position of cell bodies varied from a typical posteromedian location to a more anterolateral location in a few cases. In contrast to the CAPA-ir fibers in abdominal neuromere 2, the axons of the CAPA-ir neurons in unfused abdominal ganglia usually crossed the neuromere separately before joining in the midline of the dorsal cortex (Fig. 5A). In 2 out of 15 analyzed unfused abdominal ganglia, the bulb- or tonguelike PSOs were not located at the ventral side but at the opposite side at the anterodorsal margin of the ganglion. The terminal ganglion (with abdominal neuromeres 6–11) did not show CAPA-ir neurons. An overview of the CAPA-ir neurons in the VNC of adult *S. invicta* is given in Fig. 5.

#### 5.2 A. sexdens

**Figure 6:**
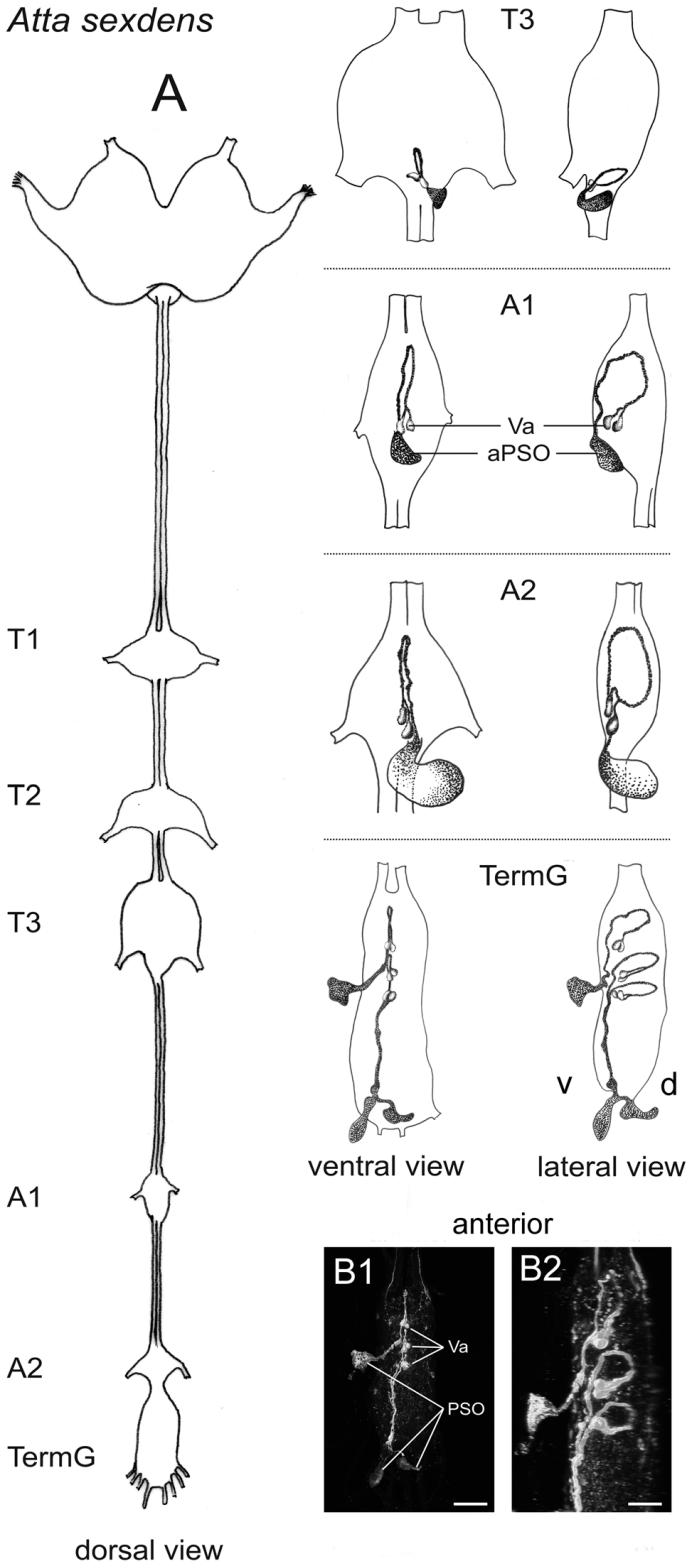
Localization of CAPA-ir neurons in the VNC of *A. sexdens*. **A**) Schematic overview of the CNS, showing CAPA-ir neurons (Va) with attached PSOs in abdominal neuromeres 2–7. Abdominal neuromeres 1 and 2 are fused with thoracic neuromere 3 in the metathoracic ganglion (T3). Therefore, the first unfused abdominal ganglion (A1) represents abdominal neuromere 3 and so on. Note the posteroventral position of the PSOs. **B**) CAPA immunostaining in the terminal ganglion; ventral (**B1**) and lateral view (**B2**). CAPA neurons are detectable in three neuromeres of the terminal ganglion; their axons always project into ventrally located PSO which position is clearly posterior of the respective neuromeres. A, abdominal ganglion; d, dorsal; PSO, perisympathetic organ; T, thoracic ganglion; TermG, terminal ganglion; v, ventral; scale bar 50 μm.

The VNC of adult *A. sexdens* consists of three thoracic ganglia with the ganglion in the metathorax composed of thoracic neuromere 3 and abdominal neuromeres 1+2, only two unfused abdominal ganglia, and the terminal ganglion. The first unfused abdominal ganglion is located in the first node of the petiole, the second abdominal ganglion is located in the gaster immediately posterior of the petiole. Immunostainings of the VNC of *A. sexdens* with the *P. americana* PVK-2 antiserum yielded six abdominal neuromeres (2–7) with CAPA-ir neurons. Somata of these cells were always located ventrally. In abdominal neuromere 2, immunostainings revealed a cell location and projection very similar to those described above for *S. invicta*. In the two unfused abdominal ganglia (abdominal neuromeres 3, 4), the initial projection of the posteromedially located CAPA-ir somata is also nearly identical with the projection of CAPA-ir cells in *S. invicta*, but the axons of the two CAPA-ir cells joined each other already before projecting dorsally. Different from the situation observed in *S. invicta*, the respective abdominal PSOs were not located in an anterior position but posteroventral. Therefore, after reaching the midline of the anteroventral cortex of the ganglion, the CAPA-ir fibers curved posteriorly and crossed the ganglia ventral to the CAPA-ir somata before entering the respective PSOs. The terminal ganglion (with abdominal neuromeres 5–11) showed three neuromeres (abdominal neuromeres 5–7) with CAPA-ir neurons. As in the unfused abdominal ganglia, the respective PSOs were clearly located posteroventrally of the CAPA-ir somata; cell location and projection were similar to those described for the unfused ganglia. An overview of the CAPA-ir neurons in the VNC of *A. sexdens* is given in Fig. 6.

#### 5.3 A. mellifera

**Figure 7:**
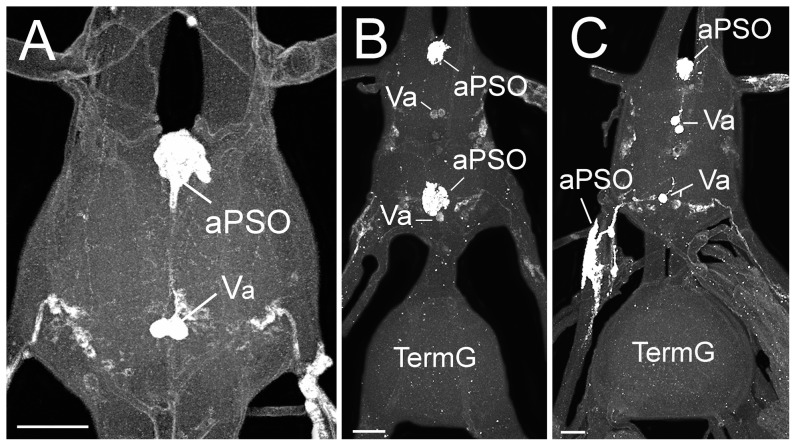
Localization of CAPA-ir neurons in abdominal ganglia of adult female honeybee, *A. mellifera*. As in *A. sexdens*, two immunoreactive ventral Va-cells are detectable in neuromeres 2–7. **A**) Va neurons in unfused abdominal ganglion 2 with axons projecting anterodorsally and leaving the ganglion into a PSO which is slightly protruded from the anterodorsal ganglionic sheath. **B**) Va neurons in fused abdominal neuromeres 6 and 7 which project into PSOs embedded in the dorsal ganglionic sheath. **C**) Example for the development of an abdominal PSO 7 that is well separated from the ganglionic sheath. Immunostaining in posterolateral cells with projection into segmental nerves cells likely resulted from cross-reactivity with unknown peptides. This was revealed by a comparison of mass spectra from abdominal PSOs and segmental nerves of unfused abdominal ganglia (not shown). PSO, perisympathetic organ; scale bar 50 μm.

The VNC of female *A. mellifera* consists of thoracic ganglion 1, a ganglionic mass consisting of thoracic neuromeres 2+3 and abdominal neuromeres 1+2, three unfused abdominal ganglia (with abdominal neuromeres 3–5), and two fused abdominal ganglia (abdominal neuromeres 6, 7) prior to the terminal ganglion (abdominal neuromeres 8–11). Immunostainings with the *P. americana* PVK-2 antiserum yielded six abdominal neuromeres (2–7) with two ventral CAPA-ir cell bodies each. Axons of these posteromedially located somata projected anterodorsally. In the dorsal midline of abdominal neuromeres 3–6, the axons then projected together or slightly separated anteriorly and innervated PSOs which were more or less protruding from the dorsal anteromedian ganglionic sheath (Fig. 7A). In neuromeres 2 and 6, the neurites of the *capa* cells enter PSOs which are usually located opposite to the somata at the dorsal ganglionic sheath. Particularly the PSO of neuromere 7 showed variability regarding shape and position along the longitudinal axis of the ganglion; ranging from the development of PSOs typical of the more anterior abdominal ganglia (Fig. 7B) to the development of well separated PSO (Fig. 7C).

## Discussion

CAPA-peptides are typically synthesized from the neuroendocrine system of the abdominal VNC in insects [10]. Expressed in at least two ventral neurosecretory cells of abdominal ganglia (Va-neurons in *Drosophila melanogaster* [12], [13], VL-cells in *P. americana* [14], NS-M4 neurons in *Manduca sexta* [15], [16]), these peptides are transported into neurohemal release sites that are usually developed as distinct abdominal PSOs [17]. The mature neuropeptide species (CAPA-PVKs and CAPA-PK) processed from the CAPA precursor were structurally first identified in the cockroach *P. americana* [18], [19]. Current available genome data, sequence similarities of processed peptides and the corresponding receptors suggest a common ancestor of *capa* and *pk* genes. Possibly within the Pancrustacea lineage, a gene duplication of this predecessor occurred which, in turn, might have a common ancestor with neuromedin U of vertebrates [20]. In insects, the *pk* gene is known by different names (e. g. *pban* of Lepidoptera, *hugin* of *Drosophila*) but is always expressed in neurosecretory neurons of the SEG [5], [21]–[25] which is different from the major expression site of the *capa* gene. CAPA-PVKs and CAPA-PK (C-terminal WFGPRLamide) both have specific receptors [26], [27].

CAPA-PVKs are known to stimulate visceral muscle contraction and regulate the activity of Malpighian tubules in many insects [10], [28]. However, the function of CAPA-PK is largely unknown. The *capa* gene was missed in recent genomic analyses of the ants *Acromyrmex echinatior* [2], *Camponotus floridanus* [29], *Harpegnathos saltator* [29], and *S. invicta* [3] and also in the parasitic wasps *Nasonia vitripennis*, *N. giraulti*, *N. longicornis* [1]. This absence raised questions about possible compensation of functions of CAPA peptides. In previous studies on *S. invicta* [5], [30], distinct PK/PBAN immunoreactivity was observed in abdominal ganglia and associated PSOs. However, *pk/pban*-mRNA transcription was not detectable in these tissues (see also Fig. 2), which suggested expression of sequence-related peptides from another gene. The candidate gene was the hitherto unidentified *capa* gene; subsequent immunostainings by means of a CAPA-PVK (PRVamide-based) antiserum demonstrated a cellular pattern identical with that obtained with the PK/PBAN antiserum [5]. The presence of these immunostainings in abdominal ganglia was thus a convincing argument to specifically search for the *capa* gene in *S. invicta*.

Our experiments demonstrated that ants possess and express a *capa* gene and alternative transcripts in the CNS. Alternative splicing of neuropeptide gene transcripts arise from differential use of splice sites localized within the gene transcript domain and skips single or multiple exons to modify the presence or sequence of mature peptides. The long and short *capa* transcripts, which were similarly abundant in the VNC of *S. invicta*, differed by 42-nt. This alternative splicing does not affect the translation of CAPA-PVK and PK peptides, but the short form of the precursor contains a truncated NPP sequence (QKLKIND; long form SVGQNYEPTREGQKLKIND). This peptide (pQ/QKLKIND) was not observed in mass spectrometric analyses. Examples of alternative mRNA transcript splicing were found in e. g. human genes [31], insect neuropeptide genes [32]–[35], and neuropeptide receptor genes [36]–[38]. However, the well established differential distribution of CAPA peptides in the CNS of *D. melanogaster* (Va-neurons/abdominal PSOs: PVKs, CAPA-PK, CPPB; SEG/retrocerebral complex: only CAPA-PK and CPPB [39]) and other insects (e. g. *Aedes aegypti*, [40], *Rhodnius prolixus* [41], *Phlebotomus papatasi* [42]) likely resulted from differential precursor processing. No evidence for multiple *capa* mRNA transcripts was found for these species.

The number of CAPA-PVK and PK peptides translated from *capa* genes of holometabolous insects is different between taxa (see Figure S2). Regarding conserved neuropeptides with substantiated receptor activation, the most significant difference between hymenopteran species and other insects is the absence of a CAPA-PK sequence in Hymnoptera with a C-terminal motif WFGPRLa. In several insect species two PKs are encoded in the *capa* gene, one with the WFGPRLa-motif and another one with a C-terminal YXPRLa-motif. Since both of these PK forms are also encoded in the *capa* gene of the cockroach [43], the presence of two forms of PKs is likely a plesiomorphic character in insects which was also observed in holometabolous insects such as *A. aegypti* [40] and *Tribolium castaneum* [44] but not in *A. mellifera* [45]. In *D. melanogaster* (and other insects; see Figure S2) the second PK with a C-terminal YXPRLa-motif has been lost and the remaining CAPA-PK belongs to the WFGPRLa-type. This PK activates, in *D. melanogaster*, a specific receptor (PK-1 receptor [26]). The *capa* gene of *S. invicta* represents the first *capa* gene identified in insects, whose sequence conserved the PK of the YXPRLa-type but lost the PK of the WFGPRLa-type. Therefore, the *S. invicta* CAPA-PK is likely not ligand of a WFGPRLa- receptor but rather activates homologs of the *D. melanogaster* PK-2 receptors [46]. A further study is needed to confirm this receptor activation. Mass spectra obtained from PSO preparations confirmed, that the *S. invicta* PK is at least partially cleaved from the C-terminal precursor. The abundance of *S. invicta* PK in abdominal PSOs was, however, much lower than the abundance observed for the PVKs.

**Figure 8:**
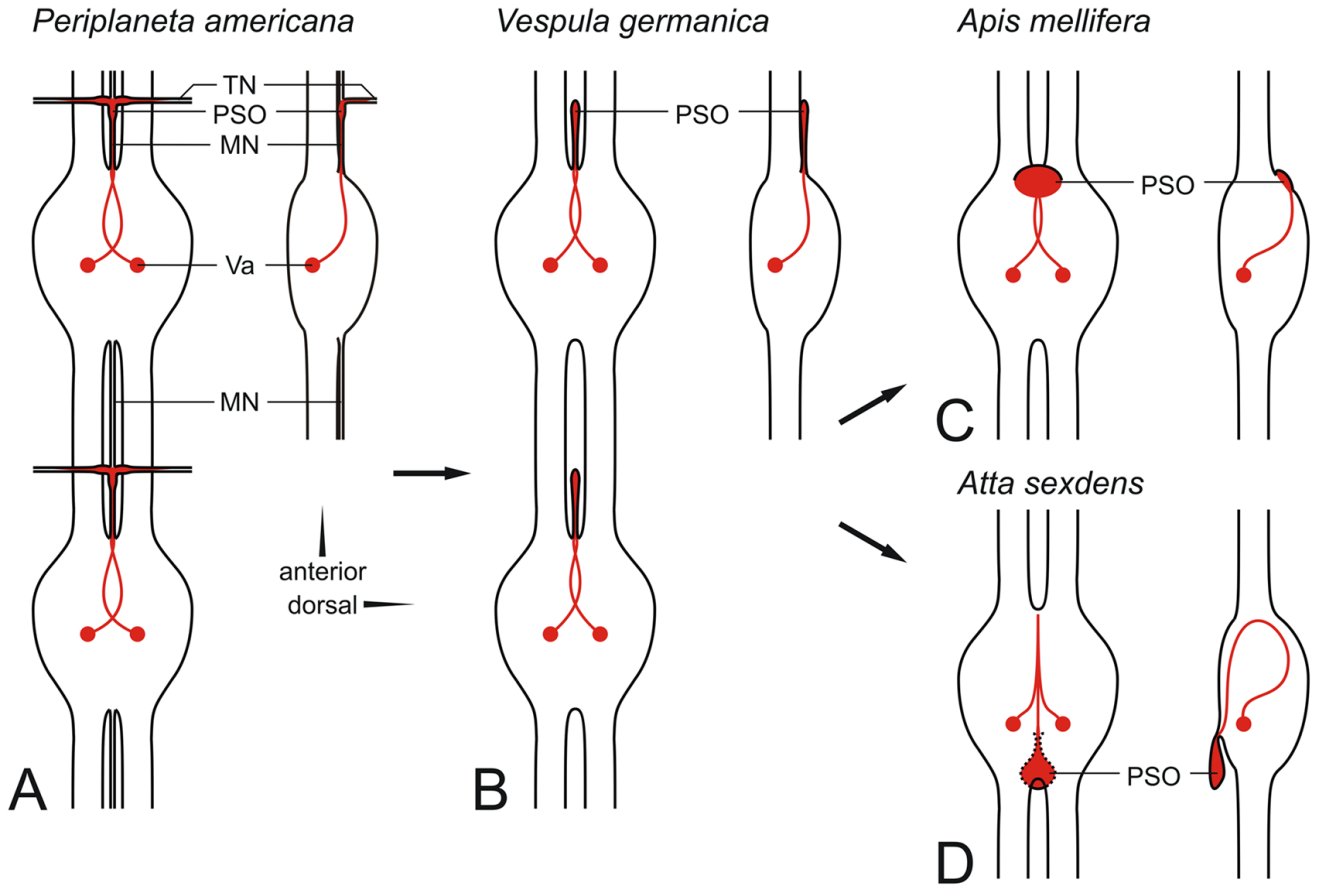
Hypothetical development of the architecture of abdominal PSOs in some hymenopteran insects. Arrows do not indicate phylogenetic lineages. **A**) Plesiomorphic situation as e. g. in the cockroach *P. americana* [17] with CAPA peptide accumulating PSOs at the junction of median nerve/transverse nerves. **B**) Intermediate development as e. g. in *Vespula germanica* (Vespidae, Aculeata); transverse nerves are no longer linked with the median nerve. CAPA peptide accumulating PSOs are club-shaped modifications of the anterior median nerve (Predel et al., unpublished). **C**) In *A. mellifera* (Apidae, Aculeata), the remains of the median nerve are more or less incorporated in the anterodorsal ganglionic sheath; the neurohemal nature of this tissue is well visible in anti-CAPA immunostainings (see Fig. 7). **D**). Ants (Formicidae, Aculeata) possess abdominal PSOs that are as distinct as in *Vespula* but their location is moved from an anterodorsal position to an anteroventral or (in *A. sexdens*) even to a posteroventral position, which is unique in insects.

The location of *capa* neurons (Va-neurons) in abdominal ganglia of *S. invicta* is typical of holometabolous insects. However, the position of abdominal PSOs, which store these peptide hormones, is unprecedented. Axons of the two ventral *capa* expressing Va-neurons first project dorsomedially into the anterodorsal midline of the respective neuromere; without showing the distinct loop around the midline that is known from many insects [10], [13]. Unlike in all other insects studied so far (including *A. mellifera* in this study), the axons then pass to the ventral side of the ganglion and enter an anteroventrally located PSO. Only in a few cases and as an obvious reversion to the plesiomorphic formation, we observed anterodorsally located PSOs at abdominal ganglia. In *S. invicta*, only four ganglia (those with abdominal neuromeres 2–5) exhibited CAPA-ir neurons. These ganglia included the three unfused abdominal ganglia but not the terminal ganglion. To test the generality of the observed features, particularly of the ventral position of abdominal PSOs, we studied with *A. sexdens* a second ant species which belongs to the same subfamily (Myrmecinae). Notably we observed not only ventrally located abdominal PSOs in *A. sexdens* but these PSOs were found invariably in a posterior position. Thus, axons of Va-neurons from *A. sexdens* first project dorsomedially into the anterodorsal midline of the neuromeres, then pass to the ventral side of the ganglia (as in *S. invicta*), curve posteriorly and cross the ganglia along the midline and ventral to the CAPA-ir somata before entering posteroventrally located PSOs. These findings suggest a position of abdominal PSOs of *S. invicta* intermediate between the original location in insects along the anterior median nerve/transverse nerve (“primitive type” of abdominal PSOs; [17]) and the highly derived posteroventral position of abdominal PSOs in *A. sexdens* (see Fig. 8). The comparison of CAPA immunostainings from *S. invicta* and *A. sexdens* revealed another interesting point with the number of segments developing Va-neurons. Whereas in *S. invicta* (3 unfused abdominal ganglia) only four neuromeres retained Va-neurons with *capa* gene expression, *A. sexdens* (2 unfused abdominal ganglia) showed six abdominal neuromeres with *capa* expressing Va-neurons. Thus, Hymenoptera not only show great variability in the number of abdominal ganglia [47] but also variability in the number of *capa* expressing neuromeres and this development does not depend on the number of unfused ganglia. From *D. melanogaster* it is known that Va-neurons are initially generated in all VNC segments before they develop a different identity anterior to the second abdominal neuromere and undergo segment-specific programmed cell death in posterior segments [48], [49]. In the two ant species studied, a similarly programmed cell death of Va-neurons could therefore affect a different number of posterior neuromeres.

In summary, this study describes *capa* gene expression, processing and localization of CAPA peptides in the VNC of *S. invicta* and demonstrates that non-detection of genes by bioinformatic analyses of genome data does not necessarily mean that the genes are absent.

## Supporting Information

Figure S1 **Nucleotide sequence and translated amino acids of fire ant** ***capa*** **gene.** *S. invicta* PVK-1, −2, −3, and PK neuropeptides (underlined) are predicted using putative endoproteolytic cleavage sites (bold italic). Alternative splicing nucleotides (42-nt; highlighted in gray) correspond to exon 2. Expected TATA box as promoter binding site, the first ATG as the initiation codon, and TAA as the termination codon are indicated with boxes.
(DOCX)

Figure S2 **Translated amino acid sequences of selected** ***capa*** **genes from holometabolous insects.** Note that the C-terminal sequence of the CAPA precursor from *A. mellifera* is different from the sequence published by Hummon *et al*. [45].
(DOCX)
