# Correction: Identification and Expression of *Capa* Gene in the Fire Ant, *Solenopsis invicta*

**DOI:** 10.1371/journal.pone.0103803

**Published:** 2014-07-23

**Authors:** The PLOS ONE Staff

There is an error in Figure 3 of the published article. The ion signal labelling is incorrect. Please view the correct figure, provided by the authors, below.

**Figure 3:**
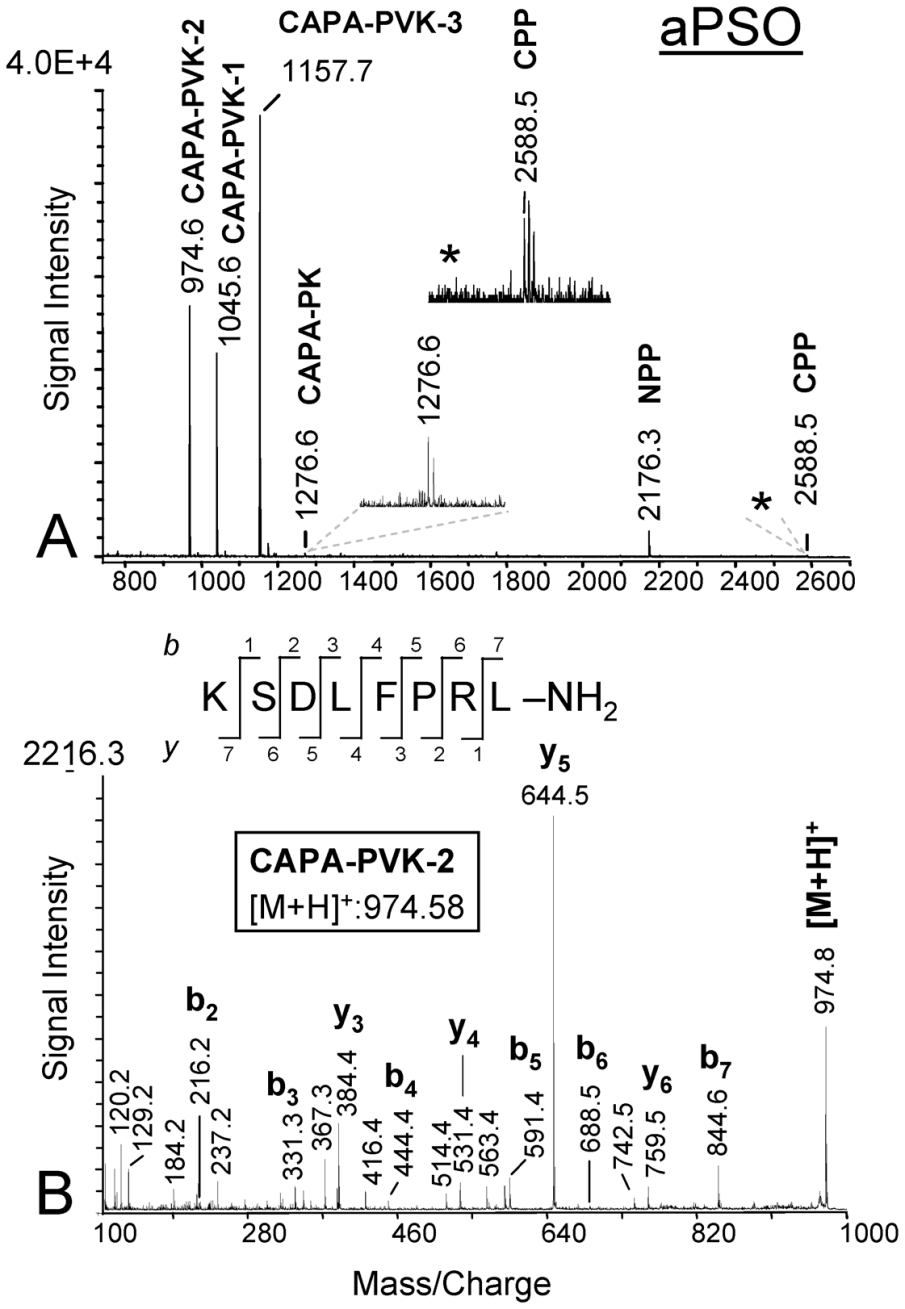
MALDI-TOF mass spectra from a preparation of a single abdominal PSO of female *S. invicta* (direct tissue profiling). A) Mass fingerprint spectrum (*m/z* 750–2700); prominent signals indicating the presence of three PVKs and the NPP (long transcript form) are detectable. The predicted PK shows a very weak signal intensity. This low signal intensity is likely a result of incomplete cleavage from the remaining C-terminus of the precursor sequence. With the exception of the supposed CPP at *m/z* 2588.5, sequences of all designated peptides were confirmed by MS/MS analyses. B) MALDI MS/MS fragment spectrum of the ion signal at *m/z* 974.6 (see 3A) under conditions of CID off. Fragment series (b- and y-type ions are labelled) confirmed the sequence of *S. invicta* PVK-2. aPSO, abdominal perisympathetic organ; PVK, periviscerokinin; PK, pyrokinin; CPP, C-terminal precursor peptide; NPP, N-terminal precursor peptide.
