# Supplementary material for: Identification and Expression of Capa Gene in the Fire Ant, Solenopsis invicta
Source: PLoS One. 2014 Apr 9;9(4):e94274. doi: 10.1371/journal.pone.0094274 (PMC3981796; doi:10.1371/journal.pone.0094274)
Supplement: Figure S1 — Nucleotide sequence and translated amino acids of fire ant capa gene. S. invicta PVK-1, −2, −3, and PK neuropeptides (underlined) are predicted using putative endoproteolytic cleavage sites (bold italic). Alternative splicing nucleotides (42-nt; highlighted in gray) correspond to exon 2. Expected TATA box as promoter binding site, the first ATG as the initiation codon, and TAA as the termination codon are indicated with boxes. (DOCX) [file pone.0094274.s001.docx]

**Figure S1**

Nucleotide sequence and translated amino acids of fire ant *capa* gene. *S. invicta* PVK-1, -2, -3, and PK neuropeptides (underlined) are predicted using putative endoproteolytic cleavage sites (bold italic). Alternative splicing nucleotides (42-nt; highlighted in gray) correspond to exon 2. Expected TATA box as promoter binding site, the first ATG as the initiation codon, and TAA as the termination codon are indicated with boxes.

-2930 ctacttctagggactctactaaaaactaaagtcaacaatatattagaaatattttggattttttcattaacttttcaataatatttctacaaggttttaagtgca

aaaatcgcggatattttctatacagaaacatctctgaaatgttgcagatatatttcgaaatgtttcgtttcttcagttgaaatctttctattttaaagcatctttgaaaaatttcggtgttgtaggggtaagacatcacgaaattacgtacatattttattgtgctaagctgatcgaaaggatgcttgtttttcatcttttcaggattaaactatctttctctcttttttttttatataatgtctaaattattggctgatgaaaagcgcaatctgcagatgcattagcagaaatttacaactttttaatccgcactactttcttggttcgaaataaattcattgaacacacgtctctttatctaggttttttttcatatccatatatctatgtacagtacacgtgtacaattaattaaacagtatgccggcgctaaacgttaaatacgatcctcatcgacgctcctttatatttaaatgagccgttatgcgcatacaaatgaaatgctcactagcagtacgtcgtgtccgtcgtggataaattaaactatgaggcagaaacccttcctttgcacccgagacaataagctaccagtttacctaattttccagtacatcgtgactatttgtaaattttatgcgtattaatcgcgtttttttatctattttttcagaaaatatttattagaaaataaaatcaccgactgaagctcataattttgtaagaaactgcagaaacattgtcattaattcttttgtttcttatgcttttgtaagaaaaattcttacggaataacttttctccgtacaaagaattatttgagaagaagagagaagtcaatttacctgcaagctagtaacatgtacaatatataaccgtaatttaatttacataatatcatcatttacacgtgtcagttatgagaaagtaatacagccataaattaaattatttatgtgttatttgaagttatgaattcataatcttcggaaaggtaaattgtgaggtaagggtctgccggtattgacatttttgccattcatttttgaagaactatgtttcacgcctctatcgaatcgaatgtatccagcgtgaccggacgtaaacctcgtgcaggaatagcgcgaaaaagcatcgcgagaacgttcacacacgttagagagattatcctcaatggaagatcgtaccacatgggaaaacgtgtcgagaaaatgtcacgcacaataatacgtcataatctgtcgcgcaataatctcatcacgcgtatataaatctatatattatcttgcatcatatcgtaatgcggcctccttttacacctacgtcgccatcgaccgaattactataatactgggtaattgatgtcgcatgcggcgtctagctggaacattcaaaagtgcgtctgaagatcaatacattttttttataaataggatggtccatccctgagggtcgcatggtgtatcaaaatcggtcatgcaaattaataatatatgtttcctatgcatgaaatagtgttatttatattgtatatgagcacagcgtataccaaaggttttcgaagattggaagtgttgtaaatattatacaattttatcaatattatatattgataaaaatgttataaaaaaaacagcttgttctcttgaaaatacacatgtataataatagaatgtattttttgtagatttacatattttttttgtatatttttataactttacatatcccatattttttaattatattttataatctcatatatcacgtgagactatttaagataattgtcgtaaggtaaatgtgatacaatataaattatttttatcattataaatgtaaaactaaattacactgaaattactgaaaagatgaaaggatttcgatgaaattattttattaatttatgtccacagtatgtaggtatttatcaagataaatagtcattataattcatcgataaattataatggataattattaattgataattatcattatctatcattgacaatattttaccacatttttactccatatgataaatagcatgtattttaatatatgctaatgtgtctttattgtatatttgattagctcattatttttgtatgaaaataattttatttgccttatagaaatttactgtaaaataaaaaaaattgttattttgtttcgaatttctaaattatgagtttgatgagattgaaataatattgatttttgacttgtaaatcttaatgacgtgtcattagacttaatagcgcatcatgcagatcacgaaaaagagatttcgttagcgttcactacataaatcgagaattaacaaaaaaaatcaatcgaaatcttctaaaaatcaatcttcaagatcgcttatctctgataggtatcttgaatcgatcttctaaagaataaatttttttgattcttgattgtacaatattctttacagcattttaagctgcagcaataaataatggtcaagctgcagcaataaataattatacttattcgaataaaaatatactgtaattactaaaaattttgtaagcttaataaaaagatcgatctttaatggaatgatttttattgacaaattgatttttaagttttcgatatttagtaatccaaaagatcaattcttcggaaagaatcgtctatctttatgacgctgcgaaaaatacaatatcggtcttacaaggagtaggggtaagacacgagacacgcggacgctctgcgacaaggggtttgaactgtggggtggcagctctgtaatcgggtggagtggcgcaaggtataaaatcggacgtcgggtaacaggagac

+1 ATTCAGTGTCGGTGCGATTCGAAGCTTCACATCGTCACAACTTAGAATTTCGATCACTGGTCACAGAA 68

ATGCAGGACAACCGGTTTTTTATCTTCGTGATCCTTCTGGCATTTTCTACATCTCTTAATC 129

MQDNRFFIFVILLAFSTSLN 20

gtgagttcacacgaatttcgcaattatcttgacaattcttaaaatatacgtttactttttttgttcttcaatcattaatgtctagtgattggtcccattaattcacaaactttaacaaaaaattttttagaagtattgcagtttttatataaaaatttttaaatgatctgttaataaaaatattaaggcacttttatgatataattttttaaacgttatctaattatttttgcaagttgtacaattgaaagttaaagcgttttgataaattaagatcggcttaaattaaccgtaagatcgattcacctggtttagatattaaacttggaatttaaattgagccttaattcaaaaagtatatatagaactgtgaaaacaattggtttaatgttacaccaaatgtcattgaggatgttcgatatcataacaattgcaaataaaaaaattagaatgataaagggcagggaacttctaatctaatattaaaagtcatagtttctattctctcgcgaataatatgttagataattcattctcattgaggccaaactgcaacttggatgcaacagctcccaatgcaaagtaaaaatgaagtaggacggggaattaaaatgcaaaaaaaacatagcatgggaaaaaaagtattatactgaactcccgcaggaaatcgttgggtgccacgagtgcgagtatagattaaattgttagggattatgatgtccgacttttgatggaaaaacgtcgacgacattctatttattttgcag 896

TTGCCAGATGCTCGGTCGGCCAGAACTACGAACCCACTAGAG 938

LARCSVGQNYEPTR 34

gcaagaaactcgcatgagtgtcgcatacatttataacttttgtgtggcatttttcaaattataaatttttatcactaattctttttctttattgttctataaatatcaaacatatatacaacaaacgttataattatcgatataaacgacgcgttttactcttttatattttctttaaacgtattttacacgtttttaaatttttgtaatattttagtgtattgctgattgtacatttaatttctttacaattattataatgcttataatgatgtcttaacataaataaattaatattaatttttatttaagcttttaatagttttaacaaagaatttagatgctttgtaatatatagttaagttttttaacactgtcacttgcaaataatttttgaaatttttcatgtttttcatttatattatattttcatttatattgtagctatttctctgtataaattatttttaaattctttatacgaaatattatacacgtatgtgtataataatgataatattgtgtataataatgtgtataataatgataaatttgataaaataatatttgatgcttagcaattaatttttatttaaataaattaatataaatttttatttaaacttttaataaaaaatatagacatttgttttttttaacaccgttacttacacatgtaaatatttttaatttttaatatattttacgtttataaatcttgtatcctttatataaattattatttttaatggctttacaaagcacgtataatagtacatgcacaaaatattgcactttaattatgtgtatcgactatgaaaatatttttagctcaataatattttataatgtcttccttttacgcgttttttttacatgctgttacattatttgcgtaattagaaaattattagatgcattagactcaaataactaattgtttacaaagaaagaagatattgtctcgtaactttcaaagagaactcagcaaaatactttcgagcacatttcatgttttaaccggcaattgtctcgcttcacgttccag 1986

AGGGTCAAAAATTGAAAATCAACGATAGACGATCTGCTGGATTGGTGGCGTATCCAAGAATCGGTCGAAAATCGGATTTGTTTCCTAGACTGGGACGTACTTT

EGQKLKINDRRSAGLVAYPRIG***R***KSDLFPRLG***R***TF

CGGAATAATACAGAAGCCCCGAGTCGGACGATCCGATGATTCGAGCTTGGGTGATTTGAATCGCTTGCACGATTTACCGGCCGATACTGATATCGAGTTCTAC

GIIQKPRVG***R***SDDSSLGDLNRLHDLPADTDIEFY

ATTACACGCGATATGGAGCCTGACGTTCTCTTAAATTTTGATTATGAAG 2241

ITRDMEPDVLLNFDYE 119

gtgtgaagaactttataaaaatcatctcagaatatgaaaaaaatgtattaaacattcacttatttcacattatatggtagattatacgaaatataatcttataacgtaagattttataaaaataatttgagtaacgttactaagaattttttcaataaattagaagagcttaacgatacaattgctgcaaaatattttagcataaacgttggatacacggatgttgctgataaatgtgtaataaacgtttaacgcatttcacgtcagatcaatatccgcgatgctgttattaattaaagcggcttttaccggattgaataaaatttatgaaaaaatacgagcgcgaaatgtatacatttgtgcgaaacaatgcagcattatgtggcatgcgcttatagtttttaatgagttgatttggacgacgtcctgcggctgatctcgcctcaggacttcgtttgacgcttttaaacgaggtgcatgcaatgaaatgtatatgaattttattacatttttgtcatttataggaattcatttgttaattgtattctcttacgtag 2798

ACTATGCAGACAAACCGATAGCATTTAAACATGCTGACAAAATTCAGAAAGACGATTCCTGGTTAATGCCCGACCATGTACGCGGATACAAGGACCCTCGTTT

DYADKPIAFKHADKIQKDDSWLMPDHVRGYKDPRF

CGCACAAAAAATCGACGATCTTCGATCGTACTATTCTATTTTGAGAG 2948

AQKIDDLRSYYSILR 169

gtatgaatataaaaggatcttatttttcactatacctttcagatgttaaatgatctatcatcgattcaaatcagatcacgatttactaatgtattatacaatagcttgcgaattagaatccacgaaagtaatcaacttttctttaaattgattttgttaaaagtaaaaaaaaataatttaaattgaaagtatttcaatttaaactcagccctctatacgatgctgagaatgcatcgttcaagagagcactaactgtacgctcgcaatgatgaacaccgacatctttaaatgttgcaggtgatattgattatattatttttgctaaattaaatattctctcggaatactcggaacacatcgcgtattgaacagatatcgactgctatacacgacgtcatattcattctttgacattatgcgaatcgcttttccataaagaagtcgcgcgtcgtctgcgcttcattaatccttaaatgcgagcaatttaagacagtccacgtgcgatttttcccgctctgcaatgtgattgatctcttttcag 3489

GTTCTCGAAATAGTCAAGGTCAAGGAGGGTACACTCCAAGACTAGGTCGCGAGAGTGAGCACGACGCTGCGAATTTCCCGTAA 3572

GSRNSQGQGGYTPRLG***R***ESEHDAANFP* 196

CTCGCCCATCTTCGATTGCGATTCGCGTCTCGCGAATTTGATGGATGTTGACTCGAGGCAAAGGAAGAATTTACTGTCAAAAAATGTCAGGAACGTAACGTGCGAGGCTTTTGATCGTTTTCTCCTATTTGCATAGGCGACCCACCTTATCCTTGCACTTTGTACGCAAGTTATGCAACACAATACATAATCTGCACTTAAA 3774

tactgcagtttataatcctacatgcaatcctctcgccttgtatcgtcacgaaatagcaattcttcggcgaagcagcccgatcttgatgtaactcggcatgctattttacccagatttctatttcgagctgctcgatccaattttaatgttcgtttttataagaacgcataatgacgagagcagaaataaatgtttgatttatatcagggctgagctctaattggtgatctgttttgtatata 4016
