# Supplementary material for: Identification and Expression of Capa Gene in the Fire Ant, Solenopsis invicta
Source: PLoS One. 2014 Apr 9;9(4):e94274. doi: 10.1371/journal.pone.0094274 (PMC3981796; doi:10.1371/journal.pone.0094274)
Supplement: Figure S2 — Translated amino acid sequences of selected capa genes from holometabolous insects. Note that the C-terminal sequence of the CAPA precursor from A. mellifera is different from the sequence published by Hummon et al. [45]. (DOCX) [file pone.0094274.s002.docx]

**Figure S2**

Translated amino acid sequences of selected *capa* genes from holometabolous insects. Note that the C-terminal sequence of the CAPA precursor from *A. mellifera* is different from the sequence published by Hummon *et al*. [45].

*Solenopsis invicta* (Hymenoptera)

MQDNRFFIFVILLAFSTSLNLARCSVGQNYEPTREGQKLKINDRRSAGLVAYPRIGRKSDLFPRL GRTFGIIQKPRVGRSDDSSLGDLNRLHDLPADTDIEFYITRDMEPDVLLNFDYEDYADKPIAFKHADKIQKDDSWLMPDHVRGKDPRFAQKIDDLRSYYSILRGSRNSQGQGGYTPRLGRESEHDAANFP

*Apis mellifera* (Hymenoptera)

MRNHLFVFLVVLSIFSVSLNRGEKLKPNMRRAFGLLTYPRIGRSNAPISNLNFNRRGVESDTDFQFYSAELDPAPDKDYEDSPAPKSLGRSMHAKHADRIPKEASWLISDRPRSSKDGSWKIDEGRSIYPFLLNSDSRNSQVNGYTPTRLDRRGNDADRILRK

*Aedes aegypti* (Diptera)

mshrfnlssdldsvsegrhkrgptvglfafprvgrsdpdllewsdaaavaaalpleladdyedypireakrqglvpfprvgrsgmnaarfywpktmmpqqqkragnsgansgmwfgprlgkranaasteikgtevytprlgrnserpqigesgdlnarsssrskledferlfrssdn

*Anopheles gambiae* (Diptera)

mlagsqakppvcvalalvllgvtvhlagaeapefesvgrvskrgptvglfafprvgrsdpelnldwessamlpletaddyedypmkemkrqglvpfprvgrsgkselamaaarywqaarnlqqqqqqqsvvkraggtgansamwfgprlgkrsrfgaaaasgsseqqqqlkaeql

*Drosophila melanogaster* (Diptera)

mksmlvhivlvifiiaefstaetdhdknrrganmglyafprvgrsdpslanslrdgleagvldgiygdasqedyneadfqkkasglvafprvgrgdaelrkwahllalqqvldkrtgpsassglwfgprlgkrsvdaksfadiskgqkeln

*Tribolium castaneum* (Coleoptera)

MKTFLIYSACVVLFCIANCQGEPKEPKRNKLASVYALTPSLRVGRRSEGTDVKRRIGKMVSFPRI GRSESNWVPDDNSYGAQRPGANSGGMWFGPRLGRVQKRSENFTPWAYIILNGEAPIIREVHYSPRL GRESEEAYEEILDSNLDVL

*Manduca sexta* (Lepidoptera)

mqsavrlvvclfllssvlggsyqsgpklrrdgvlnlypfprvgrashhtwqipindlyleydpvdkrqlyafprvgrselsllrpeqhldalqpvparrtegpgmwfgprlgrsfksdedeitiqnnnlersepelmerkkrnahln
